# The Spt10 GNAT Superfamily Protein Modulates Development, Cell Cycle Progression and Virulence in the Fungal Insect Pathogen, *Beauveria bassiana*

**DOI:** 10.3390/jof7110905

**Published:** 2021-10-26

**Authors:** Qing Cai, Juan-Juan Wang, Jia-Tao Xie, Dao-Hong Jiang, Nemat O. Keyhani

**Affiliations:** 1 College of Plant Science and Technology, Huazhong Agricultural University, Wuhan 430070, China; jiataoxie@mail.hzau.edu.cn (J.-T.X.); daohongjiang@mail.hzau.edu.cn (D.-H.J.); 2 Department of Microbiology and Cell Science, University of Florida, Bldg. 981, Museum Rd., Gainesville, FL 32611, USA; 3 School of Biological Science and Biotechnology, University of Jinan, Jinan 250022, China; wjj880414@163.com

**Keywords:** histone acetyltransferase, virulence, asexual development, cell cycle, hyphal septation, metabolism, stress response, multidrug transporter

## Abstract

Chromatin remodeling is mediated in part by post-translational acetylation/deacetylation modifications of histones. Histone acetyltransferases (HATs), e.g., members of the GNAT/MYST superfamily, activate gene transcription via promotion of euchromatin formation. Here, we characterized a GNAT family HAT, Spt10 (BbSpt10), in the environmentally and economically important fungal insect pathogen, *Beauveria bassiana.* Targeted gene knockout of *BbSpt10* resulted in impaired asexual development and morphogenesis; reduced abilities to utilize various carbon/nitrogen sources; reduced tolerance to heat, fungicides, and DNA damage stress; and attenuated virulence. The Δ*BbSpt10* mutant showed disrupted cell cycle development and abnormal hyphal septation patterns. Transcriptome analyses of wild type and Δ*BbSpt10* cells revealed the differential expression of 373 genes, including 153 downregulated and 220 upregulated genes. Bioinformatic analyses revealed downregulated genes to be enriched in pathways involved in amino acid metabolism, cellular transportation, cell type differentiation, and virulence, while upregulated genes were enriched in carbon/nitrogen metabolism, lipid metabolism, DNA process, and cell rescue, defense, and virulence. Downregulated virulence genes included hydrophobins, cellular transporters (ABC and MFS multidrug transporters) and cytochrome P450 detoxification genes. These data indicated broad effects of BbSpt10 on fungal development, multi-stress response, and virulence.

## 1. Introduction

In eukaryotic cells, genomic DNA is packaged into a highly ordered chromatin structure. The basic repeating unit of chromatin is the nucleosome, which consists of ~146-bp DNA wrapped around a histone octamer assembled from two copies of each histone H2A, H2B, H3, and H4 [1]. Changes in chromatin structure govern gene expression, with remodeling processes regulated by at least two general types of chromatin-modifying activities: (i) remodeling complexes, such as SWI/SNF, which move nucleosomes to affect chromatin structure and are dependent on energy generated by ATP hydrolysis [2], and (ii) histone modifying complexes, which modify histones in a post-translational manner, e.g., methylation, acetylation, ubiquitination [3,4].

Acetylation/deacetylation is one of the most common histone post-translational modifications, with the addition of acetyl groups to specific histone residues via the activities of histone acetyltransferases (HATs) typically correlating with gene activation (re-organization from transcriptionally repressed heterochromatin to active euchromatin), and removal of histone acetylation marks (by histone deacetylases, HDACs) resulting in DNA compaction and repression of gene expression [5,6,7,8,9]. While most acetylation of histones occurs on amino acids found on their N-terminal “tails” that extend from the DNA-nucleosome structure, a number of these modifications occurs within the globular part of the protein around which DNA is wrapped. One such modification is acetylation of lysine 56 of histone H3 (H3K56ac), which can eliminate the positive charge involved in DNA binding within the nucleosome and exert a major effect on nucleosome conformation [10,11,12]. Spt10 contains a putative histone acetyltransferase domain, as well as a His_2_-Cys_2_ zinc finger binding domain [13]. In yeast, the acetylation of histone H3K56 in nucleosomes at the core histone promoters has been showed to be dependent on the *Spt10* gene [10], which encodes a GNAT superfamily histone acetyltransferase, and *Spt10* also appears to be responsible for the targeted acetylation of histone H3 Lys9/Lys14 and of the histone H4 tail at the CUP1 (metallothionein) promoter [14], although, notably, direct HAT activity has yet to be demonstrated for Spt10p [15].

*Spt10* was originally identified as one of a set of suppressors of Ty1 transposon induced mutant phenotypes in a number of promoters [16], and has been implicated as a global regulator (co-activator) of core promoter activity, acting at or near the TATA box with its zinc-finger domain mediating cooperative binding to pairs of upstream activating sequences (UAS) [13,17,18,19]. In yeast, Spt10p is thought to be targeted to the promoters of core histone genes, as these exclusively contain dual UAS elements that are not found elsewhere in the yeast genome. Yeast null mutants (Δ*Spt10*) remain viable, but show very slow growth and impairment in cell cycle progression [20,21,22]. Spt10p appears to function through cooperation with Spt21p [23], the Hir corepressor [24,25], and the SWI/SNF ATP-dependent chromatin remodeling machine [10,25], and has been shown to affect the expression of hundreds of genes in yeast, most of which showed increased expression in the Δ*Spt10* mutant, indicating that Spt10 may act primarily as a repressor more than an activator [26]. However, it has been suggested that these effects are indirect via (direct) regulation of a far more restricted number of genes, potentially limited only to the regulation of core histone genes [27]. Regardless of the mechanism, Spt10 contributes to a cell cycle regulating oscillator complex, affects cell size via mobilization of phospholipids, and functions (cooperating with histone H2A) in the regulation of metallothionein genes [28,29,30]. An intriguing feature of Spt10 is the presence of a conserved histone acetyltransferase (HAT) domain similar to that of Gcn5 [15]. Although this domain is required for Spt10 function [23], as mentioned, direct acetyltransferase activity has yet been described for Spt10, however, Spt10 is needed for the acetylation of lysine-56 of H3 on nucleosomes found at core histone gene promoters, and this acetylation is required for cell cycle-specific gene expression [10].

Aside from work in yeast, the function(s) of *Spt10* homologs in other fungi, particularly pathogenic filamentous fungi have yet to be investigated. The insect pathogenic fungus, *Beauveria bassiana*, is one of the most widely used fungal biological pest control agents worldwide [31,32]. Amongst other factors, the insect biological control potential of *B. bassiana* depends on conidial spore production, tolerances to abiotic and biotic stresses, and the production of an array of virulence factors and secondary metabolites [33,34]. Overall, phenotypes related to development and virulence are regulated by activators involved in asexual development, cellular communication, cytokinesis, carbon/nitrogen metabolism, infection process, and dimorphic transition [35]. In *B. bassiana*, three HATs including a GNAT superfamily member (Gcn5), a MYST superfamily member (Mst2), and a P300/CBP family member (Rtt109), have previously been characterized to variously affect developmental and virulence pathways in the fungus [36,37,38]. Here, we show that the *B. bassiana Spt10* GNAT superfamily member affects a distinct set of cellular targets that include those involved in conidiation, carbon/nitrogen utilization, stress responses, blastospore development, and virulence. Critical targets involved in virulence included many kinds of cellular transporters, including cytochrome P450, ABC transporters, and MFS multidrug transporters involved in pathogenicity and resistance.

## 2. Materials and Methods

### 2.1. Bioinformatic Analysis of Spt10 in B. bassiana

The amino acid sequence of *Saccharomyces cerevisiae* Spt10 (NP_012408) was used to search homologs in the *B. bassiana ARSEF 2860* genomic database (NCBI accession: NZ_ADAH00000000) [39], and subsequently other representative fungi, including fungal pathogens of plants, insects, and humans (http://blast.ncbi.nlm.nih.gov/, accessed on 23 July 2013). Sequences were aligned with the SMART program (http://smart.embl-heidelberg.de, accessed on 23 July 2013) for structural comparison, followed by phylogenetic analysis with MEGA7 software (http://www.megasoftware.net, accessed on 23 July 2013). The molecular weight and isoelectric point of BbSpt10 were predicted online by using ExPASy-Compute pI/Mw tool (https://web.expasy.org/, accessed on 23 July 2013).

### 2.2. BbSpt10 Subcellular Localization

The coding region of *BbSpt10* was amplified from wild type cDNA using the primer pair cSpt10-F/R (Table S1) and fused to the *GFP* protein (GenBank accession code: U55763) at the BbSpt10 C-terminus using a protocol as described [36]. The fusion gene *BbSpt10::GFP* was then inserted into plasmid pAN52-*bar* (conferring phosphinothricin resistance) and integrated into the genome of the *B. bassiana* wild type strain via blastospore transformation [40]. Putative transgenic colonies expressing the fusion protein were screened by resistance to phosphinothricin (200 μg/mL), followed by microscopic observation using confocal microscopy (LSCM, FLUOVIEW FV3000, OLYMPUS). For visualization, conidia were incubated in SDB (4% glucose, 1% peptone, and 1% yeast extract) at 25 °C for 3 days with aeration at 150 rpm. Hyphal cells were collected and counter-stained with the DNA-specific Hoechst 33258 dye (Sigma, St. Louis, MO, USA) for 30 min at room temperature prior to microscopy.

### 2.3. Generation of B. bassiana Spt10 Mutants

A targeted gene deletion strain of *BbSpt10* was constructed by homologous recombination using 5′ and 3′ coding fragments (1545 and 1720 bp, respectively) flanking the *bar* selection marker, and the subsequent complemented strain (Δ*BbSpt10::BbSpt10*) was constructed via ectopic integration of the full-length *BbSpt10* sequence with ~1500 bp of 5’-flanking (promoter) region (4219 bp total) cloned into a vector containing the *sur* sulfonyl urea resistance marker, following the same strategy as described in [36]. DNA fragments were amplified from *B. bassiana* genomic DNA with paired primers as listed (Table S1) using La*Taq* DNA polymerase. Putative Δ*BbSpt10* mutant colonies were screened by *bar* meditated resistance to phosphinothricin (200 μg mL^−1^) or by *sur* resistance to chlorimuron ethyl (10 μg mL^−1^) for the Δ*BbSpt10::BbSpt10* strain. Corrected integration events were verified by PCR and Southern blotting analyses (Table S1).

### 2.4. Histone Acetylation Assays: Western Blotting

Fungal strains (wild type, Δ*BbSpt10*, and Δ*BbSpt10::BbSpt10*) were incubated in SDB for 3 d, then total protein contents were extracted to detect the protein levels of β-actin and histone H3, and the acetylation level of H3-K56 by Western blotting experiments using protocols essentially as described previously [36]. Protein concentration was quantified using the BCA Protein Assay Kit (KeyGen, Nanjing, China). Protein levels of β-actin and histone H3 were determined by probing with 2000-fold dilutions of anti-β-actin (Cell Signaling Technology, catalog # 8457) and anti-histone H3 (Abcam, catalog # ab1791) antibodies, respectively. The acetylation level of H3-K56 in the loaded samples were detected with 2000-fold dilutions of H3K56ac antibodies (Merck Millipore, catalog # 07-677-I). After primary antibody incubation and washing, blots were probed with 5000-fold dilutions of goat anti-rabbit IgG antibody (Boster, Wuhan, China) and bands were visualized by chemiluminescence detection (Amersham Biosciences, Shanghai, China). All experiments were repeated three times. Signal intensities of all blots were quantified using ImageJ software (https://imagej.nih.gov/ij/, accessed on 27 June 2018) and used to compute the ratios of acetylation levels of each histone site.

### 2.5. Phenotypic Experiments

To assess growth, aliquots (1 μL) of 1 × 10^6^ conidia mL^−1^ suspensions of each strain were spotted on agar media plates. Media included SDAY (Sabouraud dextrose agar with 1% yeast extract), CZA (3% sucrose, 0.3% NaNO_3_, 0.1% K_2_HPO_4_, 0.05% KCl, 0.05% MgSO_4_, and 0.001% FeSO_4_ plus 1.5% agar) and 16 CZA-derived media, which were prepared by omitting sucrose and/or NaNO_3_ from the media and replacing the carbon source with either glucose, fructose, lactose, maltose, trehalose, glycerol, chitin, or gelatin, and replacing the nitrogen source with either β-alanine, N-acetylglucosamine, NH_4_Cl, NaNO_2_, or NH_4_NO_3_. Plates were incubated for 8 days after inoculation at 25 °C, the diameter of each colony was measured. To assess responses to different stress causing agents, samples were spotted on the CZA plates (control), and CZA supplemented with either, (i) 0.4 M NaCl, 0.4 M KCl, or 0.6 M sorbitol for osmotic stress; (ii) H_2_O_2_ (2 mM) or menadione (0.02 mM) for oxidative stress; (iii) SDS (100 μg mL^−1^) or Congo red (10 μg mL^−1^) for cell wall perturbing stress; (iv) hydroxyurea (HU) (10 mM) or methyl methanesulfonate (MMS) (0.05%) for DNA damage stress; (v) carbendazim (CBD) (5 μg mL^−1^) or iprodione metabolite (IPM) (10 ug mL^−1^) for drug resistance analysis. The diameter of each colony was measured 8 d post-incubation at 25 °C. Relative growth inhibition of each strain by each chemical was computed as (*S*_c_–*S*_t_)/*S*_c_×100, where *S*_c_ and *S*_t_ denote the areas of control and stressed colonies, respectively.

To assess conidiation capacity, aliquots (100 μL) of 1 × 10^7^ conidia mL^−1^ suspension were spread on SDAY plates (9 cm diameter) and incubated for 9 d at 25 °C in a light/dark cycle of 12:12 h. From day 4, three plugs (5 mm diameter) were taken daily from each plate, and the conidia on each plug was harvested into 1 mL of 0.02% Tween 80 by ultrasonication. Conidial concentrations suspensions were counted using a hemocytometer and converted to # of conidia/cm^2^. In addition, aerial conidia in the 7-day-old SDAY cultures were morphologically examined via SEM and TEM, and their size and density (complexity) were quantified using the FSc and SSc readings in the flow cytometry of three samples (2 × 10^4^ conidia per sample) following our previous protocols [37]. Conidial hydrophobicity was assessed in a modified aqueous-organic system described elsewhere [41]. Cell wall width were measured from ~10 hyphal cells of each strain using ImageJ software. Conidial viability, thermotolerances, and UV-B resistance of each strain were assessed as time for 50% germination of conidia (GT_50_, h) at 25 °C, mean lethal time to kill 50% of the conidia (LT_50_, min) in response to a wet-heat stress at 45 °C, and mean lethal dose to kill 50% of the conidia (LD_50_, J cm^–2^) in response to UV-B irradiation, respectively. Experiments were essentially performed as described previously [37]. 

Insect bioassays were performed using *G. mellonella* larvae with two methods of infection. Briefly, topical bioassays were performed using ~30 larvae that were immersed for 10 s in 30 mL of a 1 × 10^7^ conidia mL^−1^ suspension for cuticular infection. For intra-hemocoel assays, 5 μL of a 1 × 10^5^ conidia mL^−1^ suspension was injected into the hemocoel of each larva. Insects were maintained at 25 °C for 10 days and examined every 12 or 24 h for survival records. Bioassay experiments were repeated three times. The mean lethal times to kill 50% of hosts (LT_50_) were estimated by Probit analyses of the time-mortality trends. In vivo produced hyphal bodies were microscopically examined in the hemolymph of the larvae 72 h after topical treatment or injection. Images for the fungal (out) growth on dead larval surfaces (4 d post-death) were taken.

Total activities of extracellular enzymes and Pr1 proteases involved in cuticle degradation and fungal virulence [42,43] were quantified as previously detailed [44]. Briefly, 100 mL aliquots of a 1 × 10^5^ conidia mL^−1^ suspension was grown in CZB containing 0.3% bovine serum albumin (for enzyme induction) and incubated with aeration at 25 °C for 4 days. The cultures were centrifuged at 1320× *g* for 1 min at 4 °C, and the total biomass (mg/mL) of each culture was quantified. To assay the activity of extracellular protease activity, 100 μL of azocasein (Sigma) solution (5 mg/mL) was mixed with 100 μL of each extract and incubated for 1 h at 37 °C, followed by termination by adding 400 μL of 10% (*w*/*v*) trichloroacetic acid. After 5 min of centrifugation at 12,000× *g*, the supernatant was transferred to 700 μL of 525 mM NaOH and the solution was read at an optical density = 442 nm (OD_442_) using a spectrophotometer. The Pr1 activity was assayed in a system of 50 μL of 1mM substrate (succinyl-(alanine)_2_-proline- phenylalanine-p-nitroanilide; Sigma), 850 μL of 15 mM Tris HCl buffer (pH 8.5), and 100 μL of each extract. The reaction was incubated for 1 h at 28 °C and terminated by addition of 250 μL of 30% acetic acid. The product was centrifuged at 1250× *g* for 5 min at 4 °C, and the supernatant read at OD_410_. One unit of enzyme activity was defined as the enzyme amount required for an increase in each OD value by 0.01 after 1 h reaction, and total activity was expressed as U mL^−1^ supernatant. 

In addition, biomass (blastospores and hyphae) level (mg/mL) and blastospore concentration were quantified from the 3-day-old submerged cultures initiated with 50 mL aliquots of a 10^6^ conidia/mL suspension in CZB and TPB, which was an amended CZB mimic to insect hemolymph by using 3% trehalose as sole carbon source and 0.5% peptone as sole nitrogen source. The two quantities were then used for the estimates of dimorphic transition rate as a reference to the speed of fungal proliferation in host haemocoel. All phenotypic data were subjected to one-way ANOVA analysis, followed by Tukey’s honestly significant difference (HSD) test.

### 2.6. Examination of Cell Size, Hyphal Septation, and Cell Cycle and Progression

Hyphae were collected from the cultures inoculated with 50 mL of 1 × 10^6^ conidia mL^−1^ SDB shaken at 150 rpm for 3 d at 25 °C, stained with calcofluor white (Sigma) for 15 min, and examined for septation pattern and cell morphology under a fluorescent microscope. Cell length and width were measured from ~50 stained hyphal cells of each strain using ImageJ software. To facilitate production of unicellular blastospores for cell cycle analysis, aliquots of 50 mL of 1 × 10^6^ conidia mL^−1^ NLB (4% glucose, 0.4% NH_4_NO_3_, 0.3% KH_2_PO_4_, and 0.3% MgSO_4_) were incubated by shaking (150 rpm) at 25°C for 3 d. Blastospores collected from NLB cultures were used to determine the G_0_/G_1_, G_2_/M, and S phases of cell cycle based on the respective readings of unduplicated (1C), duplicated (2C), and intermediate DNA concentrations from flow cytometry of three samples per strain. Blastospore size and density were also assessed with the FSc and SSc readings from the flow cytometry.

### 2.7. Transcriptional Profiling of Spt10 and Development/Septation-Required Genes

Aliquots (100 μL) of 1 × 10^7^ conidia/mL suspensions were spread on SDAY plates, and the plates were incubated for 2−7 d at 25 °C. Submerged hyphae were collected from the cultures inoculated with 50 mL of 1 × 10^6^ conidia mL^−1^ SDB and shaken at 150 rpm for 3 d at 25 °C. Total RNA from two kinds of cultures above were extracted using the RNAiso Plus Kit (TaKaRa, Dalian, China) and reversely transcribed into cDNA using the PrimeScriptH^RT^ reagent kit (TaKaRa). The resultant cDNA samples were used as templates to assess transcript levels of select genes by qPCR with paired primers designed for each gene (Tables S1 and S2). qPCR was performed using the SYBR^®^ Premix Ex Taq^TM^ (TaKaRa). Measurement of fungal 18S rRNA was used as the internal standard. The 2^−ΔΔCt^ method [45] was used to calculate relative transcript level of *Spt10* or the target genes. Each qPCR experiment included three replicates and the experiments were performed three times.

### 2.8. Transcriptomic Analysis

Three replicates of 4 d cultures of Δ*BbSpt10* and wild type were grown on cellophane overlaid SDAY plates. Cells were harvested using sterilized spoons and frozen at −80 °C. Frozen cells were sent to Personal Biotechnology Co., Ltd. (Shanghai, China) for construction and analysis of transcriptomes. Total RNA was extracted using RNA Trizol (Sigma) and mRNAs were isolated from total RNAs using magnetic oligo(dT) beads. The isolated mRNAs were fragmented into segments by using the ionic disruption method and the mRNA fragments were used as templates to synthesize the first strand cDNAs using random hexamer primers. Second-strand cDNAs were then synthesized using a cDNA Syhthesis Kit (Sigma) with the first strand cDNAs as templates. Each of the double-stranded cDNAs was purified and end repaired, and single adenines were added to the ends of the cDNA molecules. Finally, a cDNA library was constructed by adding proper adaptors to the cDNA. Samples were sequenced on an Illumina HiSeq platform. All raw reads gained by sequencing the cDNA samples were filtered to generate clean tags, which were mapped to the genome of *B. bassiana* [39] at the significant levels of log_2_(Δ*Spt10*/WT ratio) < −1 (downregulated) or > 1 (upregulated), and of false discovery rate (FDR) < 0.01. All the data were normalized as fragments per kilobase of exon per million fragments mapped (FPKM). All identified DEGs were functionally annotated with known or putative gene information in the non-redundant NCBI protein databases and subjected to FunCat category classification (http://pedant.gsf.de/, accessed on 16 July 2019). Furthermore, Kyoto Encyclopedia of Genes and Genomes (KEGG) analysis (http://www.genome.jp/kegg/, accessed on 16 July 2019) was performed to enrich the DEGS into various KEGG pathways at the significant level of *p* < 0.05. Pathogen-host interactions were analyzed on PHI-database (http://www.phi-base.org/, accessed on 16 July 2019). All transcriptomics data have been deposited to the Sequence Read Archive (SRA) on NCBI (https://www.ncbi.nlm.nih.gov/, accessed on 13 April 2021) with the dataset identifier PRJNA721731.

## 3. Results

### 3.1. Bioinformatic Analysis of B. bassiana Spt10, Construction of Deletion and Complemented Strains, Subcellular Localization, and Effects on Histone H3 K56 Acetylation 

**Figure 1:**
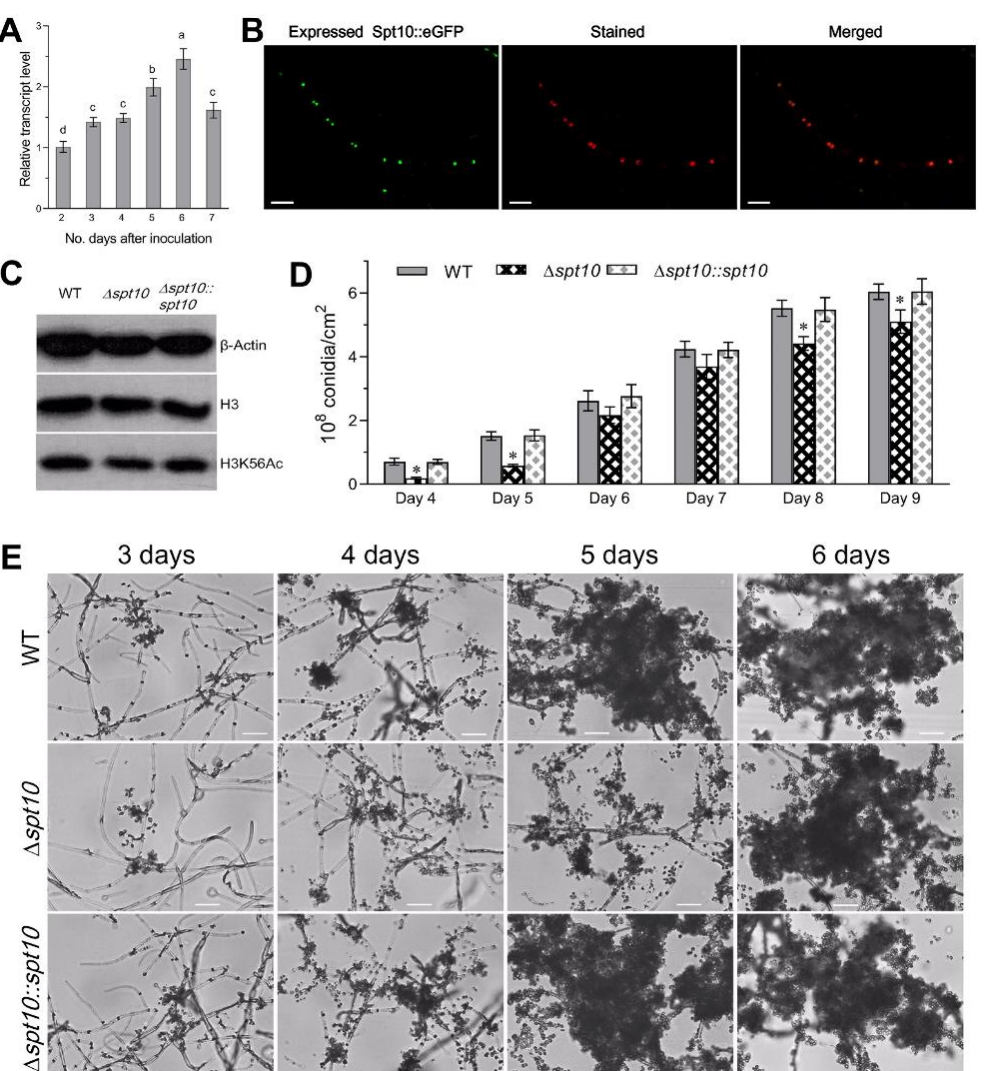
Transcriptional expression, subcelular localization of Spt10 in *B. bassiana*. (**A**) Relative transcript levels of *Spt10* in the WT cultures during 7-day incubation on SDAY at 25 °C with respect to the standard on day 2. Error bars: SD from three cDNA samples. (**B**) LSCM images (scale bars: 10 µm) for subcellular localization of GFP tagged Spt10 fusion protein expressed in WT. Hyphal cells were collected from 3-day-old SDB culture and stained with the nuclear dye Hoechst 33258. (**C**) Western blots for acetylation levels of histones H3-K56 in the protein extracts isolated from 3-day-old SDB cultures. (**D**,**E**) Conidial yields quantified or conidiation images under microscopy (scale bars: 20 µm) during normal cultivation on SDAY plates, which were spread with 100 μL aliquotes of a 10^7^ conidia mL^−1^ suspension for culture initiation. (Tukey’s HSD, * indicates *p* < 0.05).

The Spt10 homolog in *B. bassiana* (NCBI accession code: EJP69337) was identified in the *B. bassiana* genomic dataset as being encoded by a nucleotide sequence of 1944 bp containing no introns (tag locus: BBA_01302). The open reading frame (ORF) was translated into a protein consisting of 647 amino acids (molecular mass: 69.4 kDa; isoelectric point: 5.73) that contained an NAT_SF super family domain (residues 84–228) in the N-terminal domain. This is typically seen in GNAT super family members with high similarity to the Spt10 homologs found in *S. cerevisiae* (47%) and *Aspergillus nidulans* (73%) (Figure S1A), and a His_2_-Cys_2_ zinc finger domain (zf-H2C2 domain) as seen in yeast Spt10p (Figure S1A). Overall, BbSpt10 shared 34–75% sequence identity with homologs found in yeasts and other filamentous fungi (Figure S1B). Gene expression analyses indicated relatively stable expression of *BbSpt10* during vegetative growth, increasing ~2–2.5 fold during the onset of conidiation (~5–6 d, Figure 1A).

In order to characterize functional aspects of the *BbSpt10* gene, targeted gene deletion (Δ*BbSpt10*) and complemented (Δ*BbSpt10::Spt10*) strains were constructed as detailed in the Section 2, and verified via PCR and Southern blotting analyses (Figure S2, primers that were used are listed in Table S1). In addition, to investigate the subcellular localization of *BbSpt10*, a transgenic strain expressing a *BbSpt10::GFP* fusion protein was constructed. Laser scanning confocal microscopy (LSCM) of cells derived from 3 d growth in SDB showed enrichment of (green) fluorescence in the nuclei of cells (co-stained with the red fluorescence of the DNA-specific Hoechst 33258 dye) indicating the nuclear localization of BbSpt10 (Figure 1B). 

To determine the role of BbSpt10 in mediating acetylation of K56 on histones H3, protein extracts were prepared from the wild type, Δ*BbSpt10*, and Δ*BbSpt10*::*BbSpt10* strains as detailed in the Section 2. Extracts were then analyzed by Western blotting using anti-β-Actin, anti-histone H3, and anti-histone H3K56ac antibodies as probes. Densitometric quantification of Western blot signal indicated that the deletion of Spt10 resulted in a slight ~15% decrease in H3K56 bulk acetylation levels (Figure 1C). 

### 3.2. Role of Spt10 in Conidiation and Conidial Property

**Figure 2:**
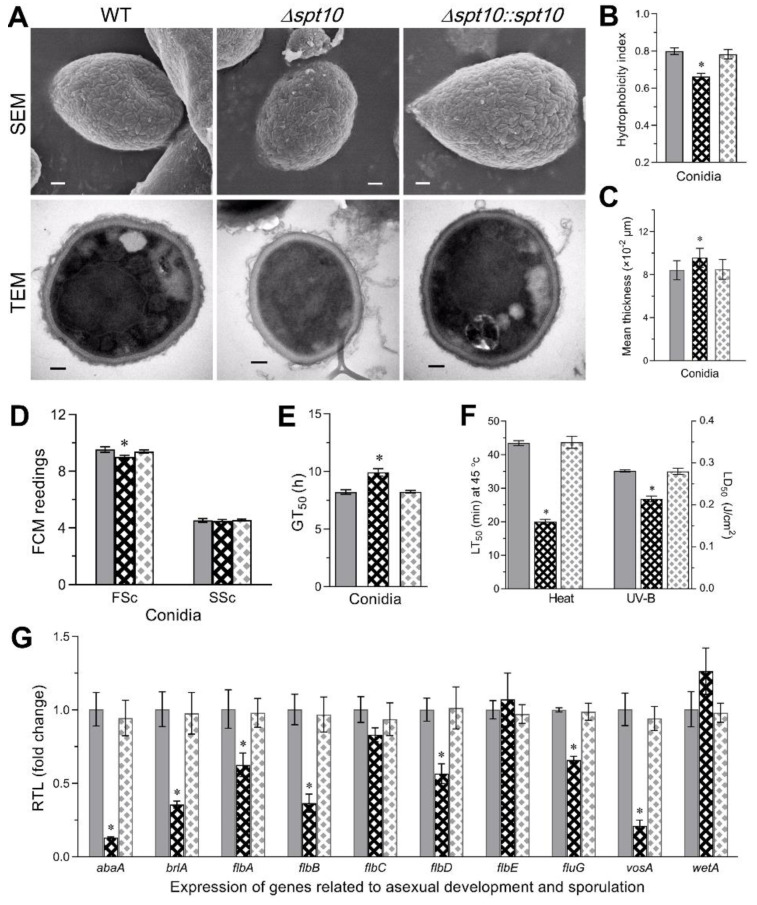
Impact of *Spt10* deletion on conidiation capacity and conidial properties. (**A**) SEM and TEM images for the conidia harvested from the 7-day-old SDAY cultures (scale bars for upper and lower panels: 0.2 μm). (**B**) Conidial hydrophobicity index quantified in an aqueous-organic system. (**C**) The mean cell wall thickness of conidia harvested from the 7-day-old SDAY cultures. (**D**) FACS analysis for cell size (FSc) and density (SSc) of aerial conidia collected from 7-day-old SDA cultures. (**E**,**F**) Conidial viability indicated by GT_50_ and conidial tolerance to heat stress and UV-B irradiation, respectively. (**G**) Relative transcript levels of 10 development-required genes in the 3-day-old SDAY cultures of *Spt10* mutants with respect to the WT standard. Asterisked bar in each three-bar group differs significantly from those unmarked (Tukey’s HSD, * indicates *p* < 0.05). Error bars: SD from three replicates.

The Δ*BbSpt10* strain showed delayed conidiation with ~62–73% decreased (*p* < 0.001) conidial yields 4 and 5 d post-inoculation of media, but only moderate (~13–20%) lower conidial yields seen from 6–9 d of vegetative growth (Figure 1D,E). Scanning electron microscopy of the conidial coat rodlet layer (where wild type conidia have been shown to contain hydrophobic fascicles or bundles on the conidial surface [46]), showed that these were disordered in Δ*BbSpt10* spores as compared to the wild type and complemented (control) strains (Figure 2A). Conidia derived from the Δ*BbSpt10* strain also showed decreased cell surface hydrophobicity, i.e., an ~20% reduction in the cell H-index (*p* < 0.001) (Figure 2B), and transmission electronic microscopy (TEM) revealed a thickening (~15%, *p* < 0.01) of the conidial cell wall (Figure 2C). 

Cell size was also slightly affected, with fluorescence activated cell sorter (FACS) analyses revealing an ~6% decrease in the conidial size of Δ*Spt10* conidia versus wild type despite no significant change in conidial density (complexity), as indicated by the readings of forward scatter (FSc) and side scatter (SSc) detectors from the flow cytometry (Figure 2D). In terms of germination, the time required for 50% of the conidia to have germinated (GT_50_) was prolonged by ~21% (*p* < 0.001) for Δ*BbSpt10* cells (Figure 2E). In terms of stress tolerances, Δ*BbSpt10* conidia were significantly more sensitive to heat stress (~54%, *p* < 0.001) and UV irradiation (~24%, *p* < 0.001) (Figure 2F).

In order to determine whether the observed reduction in conidiation was linked to the altered expression of conidiation-related genes, the expression of a suite of ten genes including (i) the *blrA* → *abaA* → *wetA*, members of the central conidiation pathway, (ii) the *flbA-E* genes (the former a regulator of G-protein signaling, and *flbD-E*, transcription factors/developmental regulators), and (iii) *fluG*, (involved in the production of a conidiation inducing signal), and the *vosA* conidial maturation factor were examined [47,48] (Figure 2G, Table S2). These data showed that expression of *BbbrlA*, *BbabaA*, and *BbvosA* was significantly (*p* < 0.001) repressed, whereas *BbwetA* expression was slightly but not significantly upregulated. In addition, all of the *Bbflb* genes with the exception of *BbflbC* and *BbflbE* were significantly (*p* < 0.01) downregulated in the Δ*BbSpt10* mutant as compared to the wild type. In all instances, here as well as below, unless otherwise stated, phenotypes of the complemented strain (Δ*BbSpt10::BbSpt10*) were indistinguishable from the wild type parent.

### 3.3. Role of Spt10 in Carbon/Nitrogen Utilization, and Multi-Stress Tolerances

**Figure 3:**
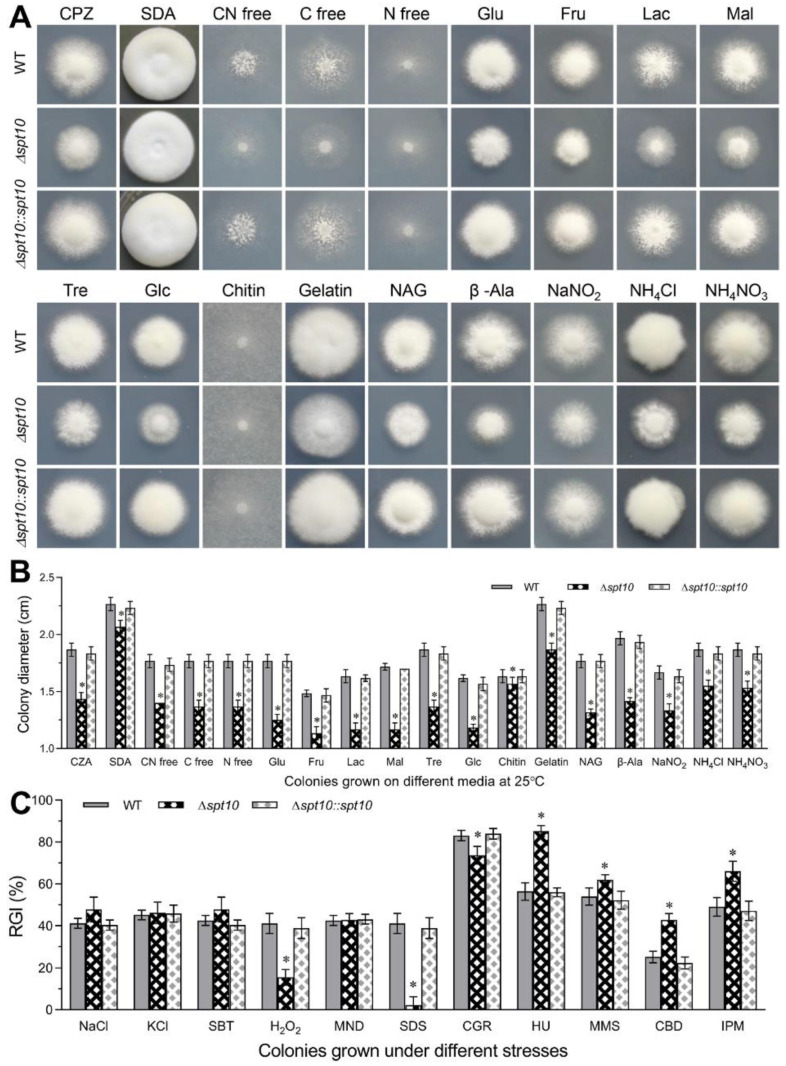
Contribution of *BbSpt10* to carbon/nitrogen utilization. (**A**,**B**) Representative images and measured colony diameters after 8 d cultivation at 25 °C on indicated media as detailed in the Section 2. Key carbon sources: Glu, glucose; Fru, fructose; Lac, lactose; Mal, maltose; Tre, trehalose; Glc, glycerol; chitin; gelatin. Key nitrogen sources: β-Alanine (β-Ala), N-Acetylglucosamine (NAG), NaNO_2_, NH_4_Cl, or NH_4_NO_3_. Additional media: carbon (C free), nitrogen (N free), or both (CN free). (**C**) Relative growth inhibition (RGI, as compared to wild type) of fungal colonies grown at 25 °C for 8 d on CZA supplemented with NaCl (0.4 M), KCl (0.4 M), sorbitol (SBT; 0.6 M), H_2_O_2_ (2 mM), menadione (MND; 0.02 mM), SDS (100 μg/mL), Congo red (CGR; 10 μg/mL), hydroxyurea (HU; 10 mM), methyl methanesulfonate (MMS, 0.05%), carbendazim (CBD, 5 μg mL^−1^), and iprodione metabolite (IPM, 10 μg mL^−1^). All colonies were initiated by spotting aliquots (1 μL) of 1 × 10^6^ conidia/mL suspension in the center of plates. All experiments were performed in triplicate. Error bars = ± SD. Asterisks indicate significantly different from unmarked (Tukey’s HSD, * indicates *p* < 0.05).

To investigate the role of *BbSpt10* in growth and development, the wild type, Δ*BbSpt10*, and complemented strains were grown in a range of media, including Sabouraud dextrose agar supplemented with yeast extract (SDAY), Czapek-Dox agar (CZA), and CZA modified with various carbon and nitrogen sources as detailed in the Section 2. Under almost all conditions tested, vegetative growth was reduced for the Δ*BbSpt10* mutant, with an ~17% decrease seen in rich media (SDAY, *p* < 0.01) and >40% decrease in growth seen on CZA (*p* < 0.001, Figure 3A,B). Similarly, minimal media (CZA minus sucrose) supplemented with eight different sugar/polyol carbon sources, including glycerol, maltose, lactose, trehalose, fructose, glucose, chitin, and gelatin, growth was decreased by anywhere from 30–54% for the Δ*BbSpt10* mutant as compared to the wild type and complemented strains (*p* < 0.001). Growth on CZA media amended with various different nitrogen sources, including on two organic (β-Alanine, N-Acetylglucosamine) and three inorganic nitrogen sources (NaNO_2_, NH_4_CL, NH_4_NO_3_), was reduced by ~31–48% for the Δ*BbSpt10* mutant as compared to the control strains (*p* < 0.001, Figure 3A,B). 

Fungal tolerances to osmotic, oxidative, cell wall perturbing agents, DNA damage stress, and fungicides were also examined. Loss of *BbSpt10* resulted in increased sensitivity to the DNA damage causing agents, hydroxyurea (HU) and methyl methanesulfonate (MMS), and to the fungicides, carbendazim (CBD) and iprodione metabolite (IPM) (Figure 3C, *p* < 0.01; Figure S3). However, no significant effects were seen in the presence of osmotic stress agents (NaCl, KCl, and sorbitol), or in the presence of the oxidative stress causing agent, menadione (MND) (Figure 3C). Interestingly, loss of *BbSpt10* resulted in decreased sensitivity, i.e., increased resistance to H_2_O_2_ and two cell wall perturbing agent, Congo red (CGR) and sodium dodecyl sulfate (SDS).

### 3.4. Impact of BbSpt10 Loss on Cell Cycle and Hyphal Septation

**Figure 4:**
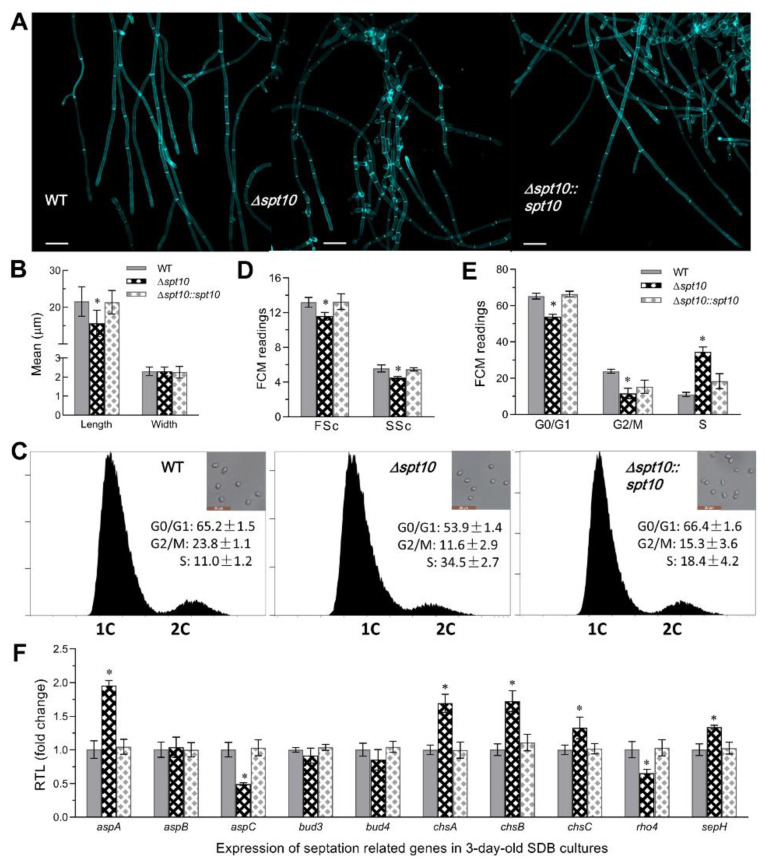
Impact of *Spt10* deletion on hyphal septation and cell cycle in *B. bassiana*. (**A**) Microscopic images (scale bars: 20 µm) for the hyphal cells collected from 72-h-old SDB cultures and stained with calcofluor white. (**B**) Mean cell length and width of hyphal cells stained with calcofluor white from each strain. (**C**,**E**) FACS analysis for distribution of cell cycle phases of blastspores collected from the liquid cultures of 10^6^ conidia mL^−1^ NLB incubated for 3 days at 25 °C. (**D**) FACS analysis for cell size (FSc) and density (SSc) of blastspores collected from 3-day-old NLB cultures. (**F**) Relative transcript levels of 10 septation-related genes in the 72-h-old SDB cultures. Asterisked bar in each three-bar group differs significantly from those unmarked (Tukey’s HSD, * indicates *p* < 0.05). Error bars: SD from three replicates.

Microscopic examination of the hyphal cells collected from the 72 h Sabouraud dextrose broth (SDB) cultures and stained with the cell wall-specific dye calcofluor white revealed altered hyphal septation patterns in the Δ*BbSpt10* mutant strain, as compared to control strains (Figure 4A). Hyphal cells of Δ*BbSpt10* were significantly shorter (~28%, *p* < 0.01) than those of control strains, but did not show any changes in terms of cell width (Figure 4A,B). In order to examine potential effects of *Spt10* on cell cycle progression, the Δ*BbSpt10* mutant and control strains were incubated for 3 days at 25 °C in nitrogen-limited broth (NLB) used to generate unicellular blastospores [40]. The resultant cells were analyzed by flow cytometry after staining with the DNA-specific dye propidium iodide. Compared to the wild type, the blastospores derived from the Δ*BbSpt10* mutant strain showed a decrease (~12%) in size and overall cell density (~19%) (Figure 4C,D). Δ*BbSpt10* derived cells showed significantly (*p* < 0.001) shorter G_0_/G_1_ (~17%) and G_2_/M (~51%) but longer S phase (~2.1 fold) times (Figure 4C,E), indicating a pronounced S phase arrest. Intriguingly, the expression of only two genes, *aspC* and *rho4*, out of ten genes examined involved in hyphal septation [49] were found to be downregulated (51% and 35%, respectively) in Δ*BbSpt10* mutant cells, as compared to the wild type and complemented strains (Figure 4F). However, expression of five hyphal septation genes, namely *aspA*, *chsA*, *chsB*, *chsC*, and *sepH*, were upregulated (23–75%) in Δ*BbSpt10*, with the expression of the three remaining genes examined, *aspB*, *bud3*, and *bud4*, being unaffected. 

### 3.5. BbSpt10 Contributes to B. bassiana Virulence

**Figure 5:**
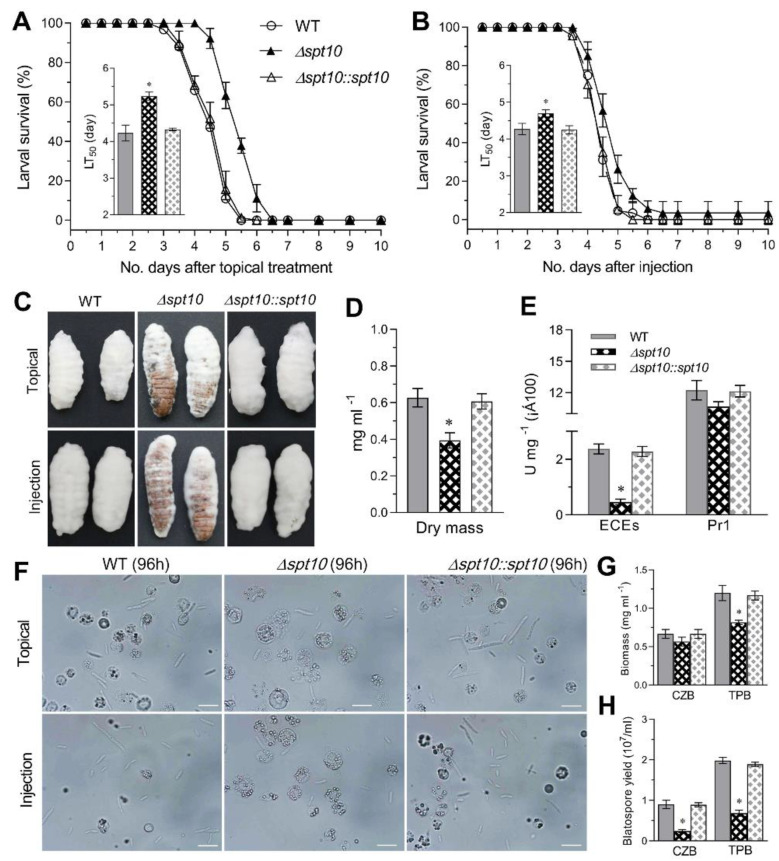
Impact of *Spt10* deletion on virulence and virulence-related properties in *B. bassiana*. (**A**,**B**) Survival trends and LT_50_ estimates of *G. mellonella* larvae after topical application of a 10^7^ conidia mL^−1^ suspension for normal cuticle infection and intrahemocoel injection of 500 conidia per larva for cuticle bypassing infection, respectively. (**C**) Images for fungal outgrowths on the surfaces of cadavers 4 days post-death. (**D**,**E**) Biomass levels and total activities of extracellular enzymes (ECEs) and Pr1 proteases quantified from the 3-day-old cultures in CZB-BSA, which contained 0.5% BSA as the sole nitrogen source for enzyme induction and 10^5^ conidia mL^−1^ for culture initiation. (**F**) Microscopic images (scale: 20 μm) for the hyphal bodies formed in the hemolymph samples of *G. mellonella* larvae 72 h post-topical treatment or post-injection. Spherical or subspherical cells represent host hemocytes. (**G**,**H**) Biomass levels and blastospore yields (dimorphic transition rates) quantified respectively from the 3-day-old submerged cultures of a10^6^ conidia mL^−1^ suspension grown in CZB and TPB at 25 °C. Asterisked bar in each three-bar group differs significantly from those unmarked (Tukey’s HSD, * indicates *p* < 0.05). Error bars: SD from three replicates.

To examine the role of BbSpt10 in affecting *B. bassiana* virulence, insect bioassays using the greater wax moth, *Galleria mellonella*, larvae as the host were conducted via both topical (representing the natural route of infection), and intra-hemocoel injection (i.e., bypassing the cuticle barrier to directly examine host innate immune responses) (Figure 5A,B). In topical bioassays, the median lethal time to kill 50% of target hosts (LT_50_) for the wild type strain and complemented strains were = 4.23–4.33 ± 0.2 d, whereas for the Δ*BbSpt10* strain the LT_50_ = 5.24 ± 0.11 d (~24% increase, *p* < 0.01). In intra-hemocoel injection assays, the wild type and complemented strain LT_50_ = 4.27–4.27 ± 0.2 d, with that for the Δ*BbSpt10* strain = 4.70 ± 0.10 d (~10% increase, *p* < 0.01). 

Visual inspection of corpses 4 d post-mortality indicated reduced fungal growth on cadavers for the Δ*BbSpt10* strain as compared to controls (Figure 5C). When cultivated on minimal CZB media, the total fungal dry mass was lower (~37% decrease) for the Δ*BbSpt10* strain as compared to the control strains (Figure 5D, *p* < 0.001), and even when normalized to this reduced growth, decreased activity in azo-casein (reflecting total extracellular proteases) (~80%, *p* < 0.001) and Pr1 protease activities (13%) (reflecting subtilisin-like proteases) were seen (Figure 5E). To further probe effects related to virulence, the development and occurrence of in vivo (within the insect hemocoel) hyphal bodies was examined. Microscopic examination of the larvae 72 h post-topical application or post intra-hemocoel injection by the control strains revealed more hyphal bodies in the hemolymph than for the Δ*BbSpt10* mutant (Figure 5F). 

These observations suggested that the transition of penetrating hyphae to the free floating (single celled) hyphal bodies was significantly delayed or impeded in the Δ*BbSpt10* mutant. To test this hypothesis, we quantified the transition rate of hyphae to unicellular blastospores for each strain after 3 d growth in CZB and trehalose-peptone broth (TPB), a modified CZB that mimics insect hemolymph. The Δ*BbSpt10* mutant showed significantly decreased total biomass production in the CZB (15%, *p* < 0.01) that was approximately doubled in the TPB media (32%, *p* < 0.001) when compared to the wild type and complemented mutants (Figure 5G). In terms of blastospore numbers (yield), the Δ*BbSpt10* mutant was even more affected, showing 65–73% decreased blastospores (*p* < 0.001) in CZB and TPB cultures (Figure 5H). 

### 3.6. Role of BbSpt10 in Global Gene Expression

**Figure 6:**
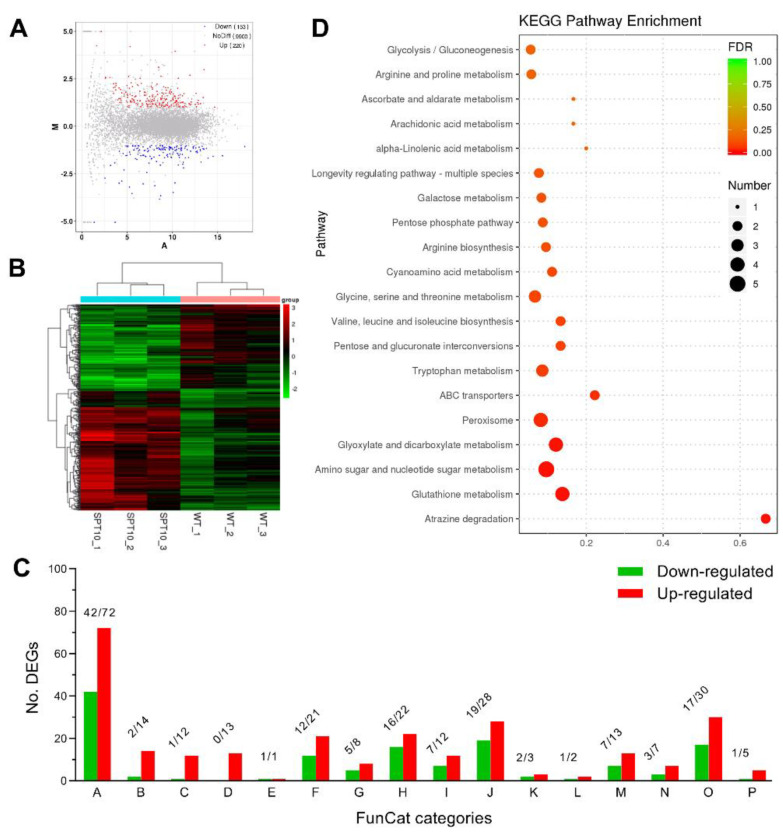
Features of Spt10-specific transcriptome in *B. bassiana*. (**A**) MA-plot analysis of the genes significantly upregulated, downregulated, and not differentially regulated (no-DEGs) in Δ*Spt10* versus WT. (**B**) Cluster analysis of differentially expressed genes (DEGs) in the transcriptomes based on the 4-day-old SDAY cultures of Δ*Spt10* and WT strains grown at 25 °C. (**C**) FunCat annotation for 16 functional categories of significantly regulated genes in Δ*Spt10* versus WT. Capital A: metabolism; B: energy; C: cell cycle and DNA processing; D: transcription; E: protein synthesis; F: protein fate (folding, modification, destination); G: cellular communication/signal transduction mechanism; H: cell rescue, defense, and virulence; I: interaction with the environment; J: cellular transportation; K: cell fate; L: development (systemic); M: biogenesis of cellular components; N: cell type differentiation; O: protein with binding function or cofactor requirement; P: regulation of metabolism and protein function. (**D**) Counts and confidencity of differentially expressed genes enriched into the top 20 KEGG pathways in Δ*Spt10*.

A comparative transcriptomic analysis using triplicate biological samples, comparing Δ*BbSpt10* and wild type cells was performed as detailed in the Section 2. A total of 373 differentially expressed genes (DEGs) were identified between the Δ*BbSpt10* mutant and wild type (Table S3 and Table S4). Of these, 153 (1.5% of the annotated genome) DEGs were downregulated (Log_2_ (ratio): −5.05 to −1.00) and 220 (2.1% of genome) were upregulated (Log_2_ (ratio): 1.00 to 4.24) in the Δ*BbSpt10* mutant, as compared to the wild type (Figure 6A,B). Approximately 46% of the DEGs could be classified into 16 functional classes via FunCat category classification (Figure 6C). Among the classified DEGs (that include DEGs distributed into multiple categories), 67% were involved in metabolism, followed by 28% in binding or cofactor requirement; 27% in cellular transportation; 23% in cell rescue, defense, and virulence; 19% in protein fate; 12% in biogenesis of cellular components; 11% in interactions with the environment; 9.4% in energy; 7.6% in cell cycle and DNA processing; 7.6% in transcription; 7.6% in cellular communication/signal transduction mechanism; 5.9% in cell type differentiation; 3.5% in regulation of metabolism and protein function; and <3% in cell fate, development (systemic), or protein synthesis (Figure 6C). With respect to histone genes, in the RNA-seq analyses, deletion of *BbSpt10* had no significant effect on the expression of histone H1 or the four core histone genes including H2A, H2B, H3, and H4.

Among those genes differentially expressed in Δ*Spt10*, eighteen genes (Table S5) were found to participate in DNA processing, transcription, and cell cycle control. Apart from one tousled-like kinase that was downregulated, seventeen genes were upregulated. For instance, six upregulated genes were involved in DNA replication and recombination including an UV-endonuclease, UvdE. There were seven upregulated genes involved in cell cycle control and cytokinesis, including a mitogen-activated protein kinase Sty1 and a chitinase-like protein. Twelve upregulated genes were involved in transcription process, including a bZIP transcription factor and Caf16, a subunit of CCR4-Not complex (a key regulatory complex of gene transcription and cytoplasmic mRNA degradation) [50].

There were fourteen differentially expressed genes (Table S6) involved in cell growth, fungal development, and cell type differentiation. Five genes were downregulated including an inversin (INVS) protein involved in Wnt/JNK signaling and cell morphogenesis [51], and a tousled-like kinase related to cell fate and polarization [52]. Nine genes were upregulated, including a β-1,3-glucanosyltransferase involved in cell wall biogenesis and vegetative fungal growth.

More importantly, there were 31 differentially expressed genes found to be involved in cell rescue, defense, and virulence (Table S7). In terms of the multi-stress tolerance, two genes were downregulated including a di-hydro-flavonal-4-reductase protein and a Hsp70 family chaperone, while eight genes were upregulated including catalase A, catalase C, and two OsmC-like proteins. Twenty-seven DEGs were related to virulence or defense, for instance, thirteen genes were downregulated including an aminotriazole resistance protein, a multidrug resistance protein 1, two ABC (ATP binding cassette) multidrug transporters, two cytochrome P450s (CYP450), and two TfdA family taurine catabolism dioxygenase TauD. Fourteen genes were upregulated, including the cuticle-degrading protease bassiasin I precursor, an SCP-like extracellular protein, an ABC multidrug transporter, a cytochrome P450, and three MFS (major facilitator superfamily) multidrug transporters.

Analysis using the pathogen-host interaction (PHI) database revealed 41 differentially expressed genes (Table S8) when comparing the Δ*Spt10* mutant to wild type, all of which are known to contribute significantly to virulence in *B. bassiana* [35]. Among these DEGs, there were 16 downregulated genes involved in reduced virulence or unaffected pathogenicity, including hydophobin Hyd2 and a fungal specific transcription factor. While twenty-five upregulated genes were involved in a loss of pathogenicity, reduced virulence, or unaffected pathogenicity, including two catalases (catA and catC) and many kinds of transporters, such as ABC-2 type transporter, a phosphate transporter, a hexose transporter, and a plasma membrane zinc ion transporter.

Aside from the FunCat classification, 67 DEGs were significantly enriched into 39 KEGG pathways (detailed in Table S9). Among the top 20 enriched KEGG pathways (Figure 6D), many DEGs were classified as the metabolism pathways of carbohydrates (amino sugar/nucleotide sugar, glyoxylate/dicarboxylate, pentose/glucuronate, galactose, ascorbate/aldarate metabolism, glycolysis/gluconeogenesis, etc.), amino acid metabolism (tryptophan, valine, leucine/isoleucine, glycine, serine/threonine, arginine, and proline metabolism), and lipid metabolism (alpha-linolenic acid and arachidonic acid metabolism), providing further insights into the growth defects of Δ*Spt10* on the tested carbon and nitrogen sources (Figure 6D).

Among the top 20 downregulated genes in Δ*Spt10* versus WT (Table S10), a fungal zinc cluster transcription factor, a beta-1,3-glucan binding protein, a GPI anchored protein, and a CFEM domain-containing protein were identified. More importantly, two critical targets were a Ncp1-like protein involved in cell cycle progression and cell polarity [53], and a Mei5 protein involved in DNA repair and meiotic recombination [54]. As for the top 20 upregulated genes, apart from several genes involved in metabolism and energy utilization, critical targets were three cellular transporters and three methyltransferases, which indicated possible cross-talks between histone methylation and acetylation mediated by BbSpt10. Interestingly, eleven downregulated genes and four upregulated genes were uncharacterized genes, which indicated significant room for further exploration.

## 4. Discussion

Protein acetylation/deacetylation has been suggested as a point of confluence for control of fungal infection [55]. Enzymes that mediate lysine acetylation (histone acetyltransferases, HATs, and more generally, lysine acetyltransferases, KATs) have been implicated in the control of key determinants important for virulence in animal and human pathogenic fungi, including *Candida albicans*, *Aspergillus flavus*, and *Cryptococcus neoformans*, and in a range of plant pathogenic fungi, such as *Fusarium fujikuroi,* and *Ustilago maydis* [56,57,58,59,60]. In *B. bassiana*, the contributions of a range of HATs and histone deacetylases (HDACs) have been investigated with reference to growth and virulence. These include the three major HATs; i.e., the GNAT superfamily member, Gcn5, the MYST superfamily member, Mst2, and the P300/CBP family member, Rtt109, as well as three HDACs, including a Class I HDAC, Rpd3, a Class II HDAC, Hos2, and a sirtuin family member, Sir2, all of which have been shown to exert various levels of contributions to cell morphogenesis and virulence [36,37,38,61,62,63]. However, Spt10, a GNAT superfamily HAT, is unique in that its substrates remain obscure and its effects in filamentous fungi, particularly with respect to the development and virulence have yet to be investigated. In yeast, loss of *Spt10* results in severe growth defects that include arrested cell cycle progression, likely linked to mis-regulation of core histone expression that can be toxic when occurring outside of the S phase [28]. 

Here, we showed that *B. bassiana* contains a single Spt10 homolog, which, as expected, was mainly localized in the cell nucleus. Western blotting revealed that deletion of *BbSpt10* resulted in a small (~15%) decrease in bulk acetylation of K56 on histone H3, similar to results seen in *S. cerevisiae*, where either no effects or small decreases (~15%) in H3K56 acetylation have been noted due to the observation that only a subset of H3 histones, namely those at core histone promoters are affected [10,64,65]. In yeast, Spt10 has been shown to have broad effects in terms of global gene transcription [17,18,19]. However, Spt10p has been suggested to be mainly, if not exclusively, found at histone core gene promoters, and that Spt10p acts indirectly on other genes via direct regulation of target histone genes. In *B. bassiana*, the expression of a moderate number of genes (373) were noted, and the overall growth defect phenotype was much more limited than what has been reported in yeast. Slightly more upregulated than downregulated genes were identified in the *BbSpt10* mutant strain, suggesting a suppressor role of BbSpt10, which is similar to findings reported in *S. cerevisiae* [19,26].

Conidia are typically the active ingredient used in insect biological control applications. Using *B. bassiana* and conidial viability is a critical parameter in mediating pest control [33]. In the Δ*BbSpt10* mutant, our data show that transcript levels of *flbA/B/D* that are required for the *brlA* activation were greatly reduced, as was the expression of *brlA*, *abaA*, and *vosA*. These data are consistent with the delayed conidiation seen for the mutant and the lowered conidial yield. This delay was likely due to the significantly longer S phase seen for Δ*BbSpt10* cells, as ordered cell cycle and hyphal septation are known to be critical for both vegetative growth and spore development [49,66]. Our data showed that loss of *BbSpt10* resulted in smaller blastospores, shortened hyphal cells, which again could be attributed at least in part to the arrested cell cycle phenotype seen in the mutant as compared to the wild type parent. These results indicate that *BbSpt10* likely affects hyphal septation and cytokinesis via cell cycle disruption. Accordingly, expression of the gene critical for septation and cytokinesis, namely *rho4* and the septin *aspC* [67,68], were significantly decreased in the Δ*BbSpt10* mutant. However, the transcription of the septins, *aspC* and *sepH*, as well as the three major chitin synthases, *chsA*, *chsB*, *chsC*, were upregulated in Δ*BbSpt10* mutant, suggesting compensatory effects that ultimately results in much milder growth phenotypes seen for *B. bassiana* as compared to yeast. 

Our data also indicate that the *B. bassiana* Spt10 is required for full virulence in topical bioassays, i.e., for the ability of the fungus to breach the insect cuticle, but has limited consequences for subsequent growth and immune evasion once inside the host hemocoel. In terms of cuticle penetration, the Δ*BbSpt10* strain exhibited decreased secretion of cuticle-degrading enzymes, including the extracellular proteases and subtilisin-like proteases. In addition, the expression of several genes known to be important for topical infection, i.e., cuticle penetration, were also shown to be negatively affected in the Δ*BbSpt10* mutant. The expression of the Hyd2 hydrophobin important for conidial hydrophobicity and required for adhesion to the host cuticle [46], was significantly decreased in the Δ*BbSpt10* mutant. In addition, the expression of a number of oxidative stress suppressing enzymes involved in overcoming host reactive oxygen species (ROS) defense responses was reduced. These included a number of catalases, superoxide dismutase, peroxidase, and peroxidase/catalase, several of which are known to contribute to virulence [69,70,71], as well as a dihydroflavonal-4-reductase (DFR) involved in cellular ROS modulation and abiotic stress tolerance [72]. However, compensatory effects were also seen with respect to oxidative stress enzymes, with upregulation of the expression of the catA and catC catalases, which may help account for the enhanced tolerance to H_2_O_2_ seen for the *BbSpt10* mutant as compared to the wild type parent. Conversely, the expression of a heat shock protein, Hsp70 family chaperone, implicated in conidial tolerance to heat stress [73] was significantly downregulated in the mutant, likely helping to account for the significant heat sensitive phenotype seen for the Δ*BbSpt10* mutant. Thus, the effects on virulence are likely due to (1) impairment in cell cycle progression, and (2) reduced expression of a subset of virulence related genes. In conclusion, our data delineate functional aspects of *BbSpt10* with heat sensitivity, impaired growth in minimal media carbon sources, delayed cell cycle progression and conidiation, changes in cell wall structure, and impaired virulence as the major consequences of *BbSpt10* loss.

## 5. Conclusions

Using the *B. bassiana* insect host-pathogen system, the GNAT superfamily histone acetyltransferase Spt10 was shown to regulate a large array of downstream gene targets that impact a wide range of physiological processes, including asexual development, cell cycle control, stress response, metabolism, and pathogenesis. In addition, at least 118 targets belonging to uncharacterized proteins were identified, indicating significant room for further exploration. Despite the large range of targets, directed regulation of hydrophobins, multidrug transporters, and other pathogenicity related proteins at the transcription levels suggest a specific mechanism(s) by which Spt10 controls resistance and virulence. These data indicate an expanded gene target set for GNAT superfamily HAT Spt10 mediated transcription control and their effects on gene expression.

## Data Availability

All transcriptomics data have been deposited to the Sequence Read Archive (SRA) on NCBI (https://www.ncbi.nlm.nih.gov/, accessed on 13 April 2021) with the dataset identifier PRJNA721731.

## Supplementary Materials

The following are available online at https://www.mdpi.com/article/10.3390/jof7110905/s1, Figure S1: Phylogenetic relationship of *B. bassiana* Spt10 with the homologs found in other representative organisms. Figure S2: Generation and identification of *B. bassiana Spt10* mutants. Figure S3: Multiple stress responses of *B. bassiana* in absence of *Spt10*. Table S1: Paired primers designed for manipulation of *Spt10* and identification of its mutants in *B. bassiana*. Table S2: Paired primers used for transcriptional profiling of potential Spt10-mediated genes in *B. bassiana* via qPCR. Table S3: Summary of 373 significantly regulated genes in *B. bassiana* Δ*Spt10* mutant versus WT. Table S4: The 373 differentially expressed genes in the heatmap of Δ*Spt10* versus WT. Table S5: Summary of eighteen Spt10-regulated genes involved in DNA events, cell cycle, and transcription in *B. bassiana*. Table S6: Summary of fourteen Spt10-regulated genes involved in cell type differentiation and morphogenesis of *B. bassiana*. Table S7: Summary of 31 Spt10-regulated genes involved in cell rescue, defense, and virulence in Δ*BbSpt10*. Table S8: Summary of 41 Spt10-regulated genes enriched in pathogen-host interaction (PHI) network in *B. bassiana* Δ*Spt10* mutant versus WT. Table S9: Summary of 67 DEGs enriched in 39 KEGG pathways in *B. bassiana* Δ*Spt10* mutant versus WT. Table S10: Summary of the top 20 upregulated genes and the top 20 downregulated genes in Δ*Spt10* versus WT.
