# Supplementary material for: The Spt10 GNAT Superfamily Protein Modulates Development, Cell Cycle Progression and Virulence in the Fungal Insect Pathogen, Beauveria bassiana
Source: J Fungi (Basel). 2021 Oct 26;7(11):905. doi: 10.3390/jof7110905 (PMC8619123; doi:10.3390/jof7110905)
Supplement: Supplementary file 1 [file jof-07-00905-s001.zip › Supporting Information.pdf]

Supporting Information

The Spt10 GNAT superfamily protein modulates development, cell cycle progression and virulence in the fungal insect pathogen, *Beauveria bassiana*

Qing Cai [1,2,*], Juan-Juan Wang [3], Jia-Tao Xie [1], Dao-Hong Jiang [1], Nemat O. Keyhani [2,*]

[1] State Key Laboratory of Agricultural Microbiology, College of Plant Science and Technology, Huazhong Agricultural University, Wuhan, Hubei, 430070, China

[2] Department of Microbiology and Cell Science, University of Florida, Bldg. 981, Museum Rd., Gainesville, FL, 32611, USA

[3] School of Biological Science and Biotechnology, University of Jinan, Jinan, Shandong, 250022, China

.

*** Corresponding author:** Qing Cai, Huazhong Agricultural University, Wuhan, Hubei, 430070, China. Phone and fax: (+) 86-027-87280487. E-mail: caiqing@mail.hzau.edu.cn; Nemat O. Keyhani, University of Florida, Gainesville, Florida, 32611, USA. E-mail: keyhani@ufl.edu.

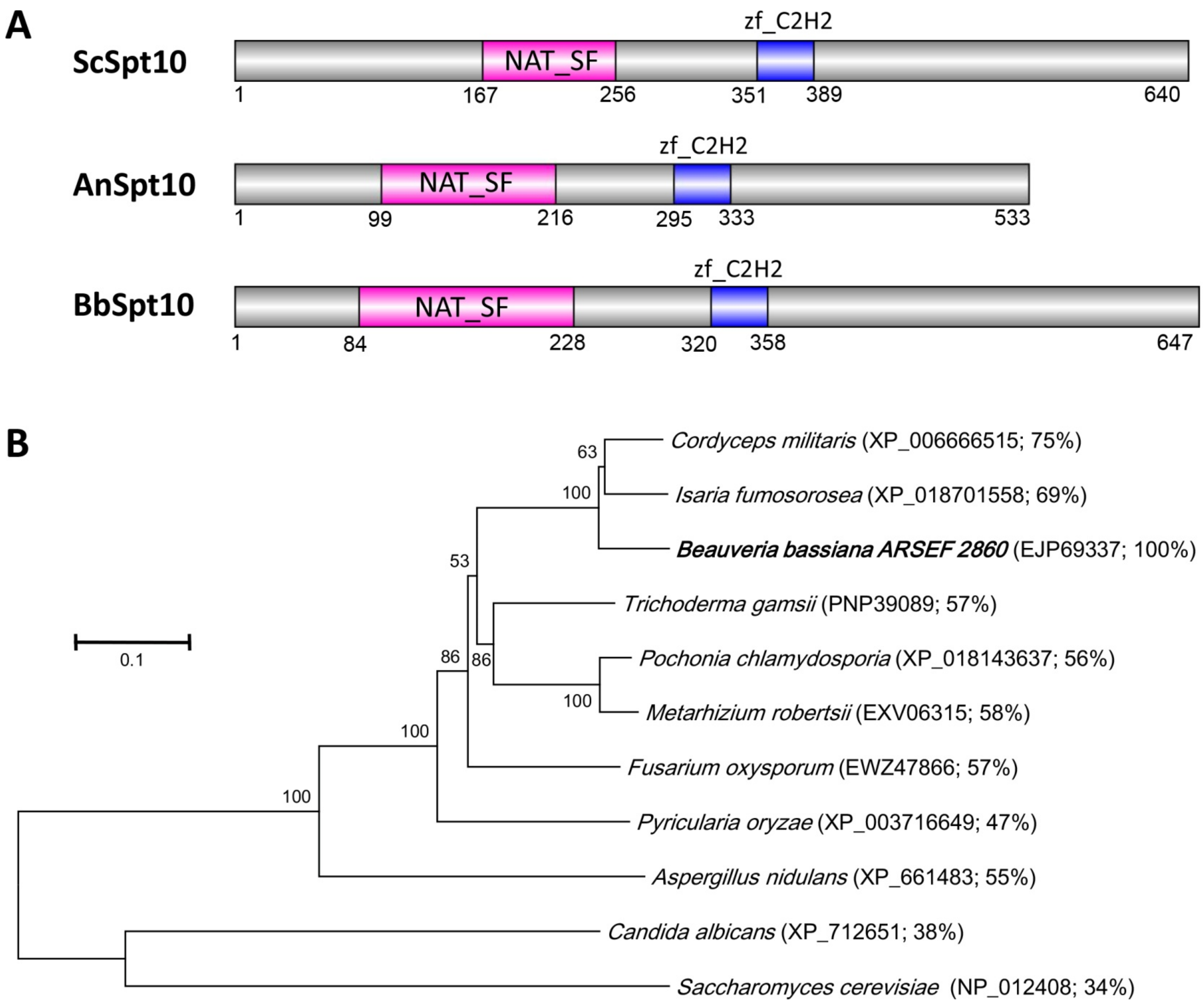

Figure S1. Bioinformatic properties of fungal Spt10 homologues. (**A**) Sequence features of Spt10 homologs found in *Beauveria bassiana* (Bb), *Saccharomyces cerevisiae* (Sc), and *Aspergillus nidulans* (An). The domain of each protein was predicted at https://blast.ncbi.nlm.nih.gov/Blast.cgi. (**B**) Phylogenetic relationship of *B. bassiana* Spt10 with the homologs found in other representative fungi. A neighbor-joining method in MEGA7 at http://www.megasoftware.net was used in the phylogenetic analysis. Each fungal name is followed by the NCBI accession code of each protein and its sequence identity (%) to *B. bassiana* Spt10 in parentheses. Poisson model was used with 1000 bootstrap replications in uniform rates. Scale bar: branch length proportional to genetic distance assessed with the neighbor-joining method.

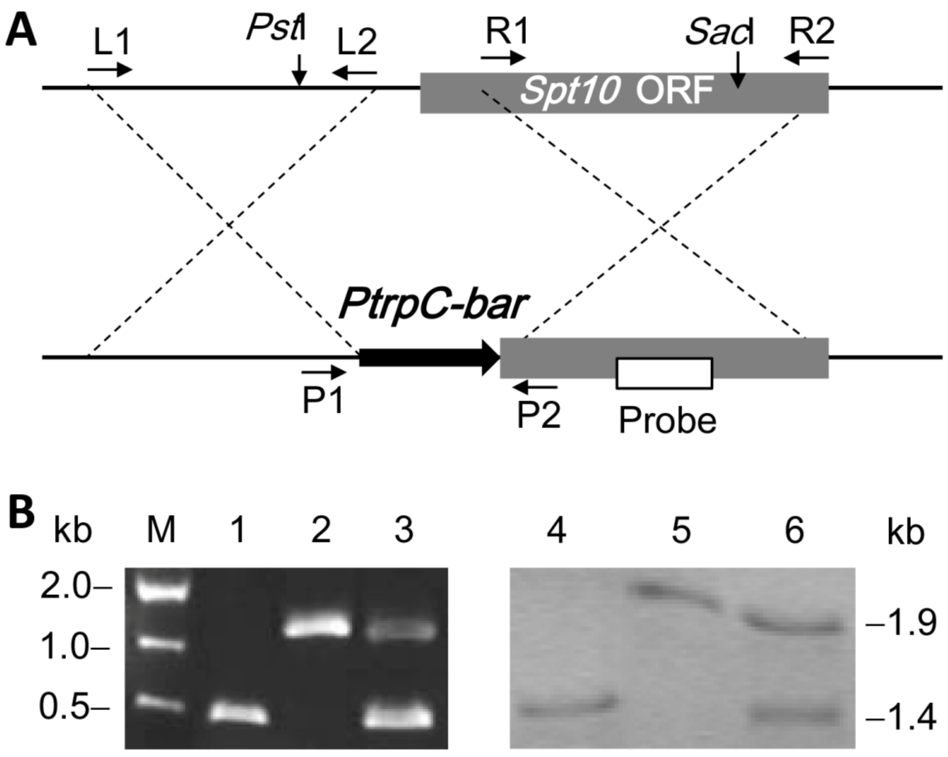

Figure S2. Generation and identification of *B. bassiana spt10* mutants. (**A**) Schematic diagram for the strategy of *spt10* deletion. (**B**) The *spt10* mutants identified via PCR (lanes 1–3) and Southern blotting (lanes 4–6) analyses with paired primers and amplified probe (Table S1). Lanes 1 and 4: wild-type. Lanes 2 and 5: Δ*spt10* mutant. Lanes 3 and 6: Δ*spt10::spt10* mutant. Genomic DNAs were digested with *Pst*I/*Sac*I at the marked sites for the Southern blotting hybridization of *spt10*.

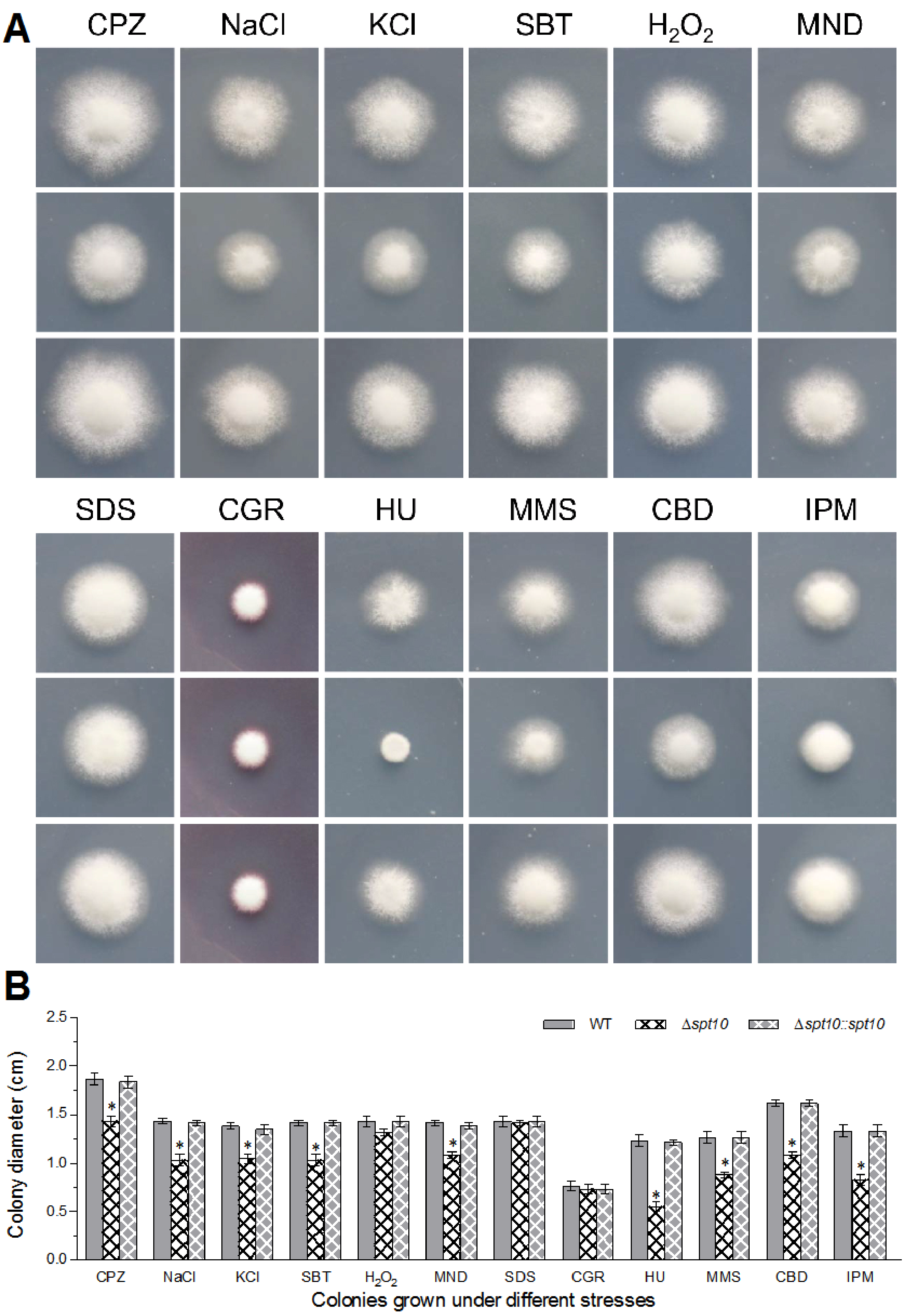

Figure S3. Multiple stress responses of *B. bassiana* in absence of *Spt10*. (**A, B**) Images and colony diameters of fungal colonies grown at 25°C for 8 d on CZA supplemented with NaCl (0.4 M), KCl (0.4 M), sorbitol (SBT; 0.6 M), H_2O_2 (2 mM), menadione (MND; 0.02 mM), SDS (0.1 mg/ml), congo red (CGR; 10 µg/ml), hydroxyurea (HU; 10mM), methyl methanesulfonate (MMS, 0.05%), carbendazim (CBD, 5 µg ml^{-1}) and ipodione metabolite (IPM, 10 µg ml^{-1}). The asteristed bar in each three-bar group differ significantly from those unmarked (Tukey's HSD, $p < 0.05$). Error bars: SD of the mean from three replicates.

Table S1. Paired primers designed for manipulation of *spt10* and identification of its mutants in *B. bassiana*.

Primers	Paired sequences (5'-3')*	Purpose
Spt10-F/R	ACCTTCAAACCCGGGGATCCATGCCTGCCGCCGTCGA/ATCTGCAGGTCGACGGATCCAGTCCACCCCCTTGTAT	Cloning *spt10* cDNA(1941 bp)
Spt10up-F/R	AAAAAGAATTCCAAGACAGGCAAAGAGGATTCA / AAAAAAAGCTTCCAGCCAGTACAACACTTGCTT	Cloning *spt10* 5′-end (1545 bp)
Spt10dn-F/R	AAAAAACTAGTATCACCTATCTCTGCGGGCA / AAAAAGTTAACCATCCAAATCGTCCATGTCC	Cloning *spt10* 3′-end (1720 bp)
Spt10fl-F/R	ggggACCACTTTGTACAAGAAAGCTTCCCAAGACAGGCAAAGAGG / ggggACAAGTTTGTACAAAAAAGCAAACATTCAGCACAAGGAGCAA	Cloning full-length *spt10* (4219 bp)
pSpt10-F/R	CGGTGTAAGTCAATACGGCAAG / CCAAAGTTTTGGAACCAGTACG	PCR detecting *spt10*
sbSpt10-F/R	GCGTCTTCAACTTGGTGT / CCGTAGTCTTGTTTATGCC	Southern blotting *spt10* (462 bp)
qSpt10-F/R	GCTCATCACCTATCTCTG / GTAAAGGGCTCCATCATC	qPCR detecting *spt10*
q18S-F/R	TGGTTTCTAGGACCGCCGTAA / CCTTGGCAAATGCTTTCGC	qPCR detecting 18S RNA

* Underlined regions denote the restriction enzyme sites used for cloning *spt10* cDNA (*BamH*I/*BamH*I) or deleting *spt10* (*Eco*RI/HindIII and *Spe*I/*Hpa*I) and the fragments of gateway exchange for *Spt10* complementation.

Table S2. Paired primers used for transcriptional profiling of potential Spt10-targeted genes in *B. bassiana* via qPCR.

Gene	Tag loci*	Annotation	Sequences (5′-3′) of paired primers
**Involved in asexual development**			
fluG	BBA_04942	Developmental protein FluG	CCTCCCTAGTTTGGTCGCTTTCTC / CGCTGTCGGTAATCTGCTCCTC
flbA	BBA_02968	Developmental regulator FlbA	CCAATCCACTCGCCGCTCTC / CGGAGGAAAGAGAATCGGTAGAGG
flbB	BBA_06988	bZIP transcription factor FlbB	GCACTGACACGCCGACAAGAGC / CCGCCGCCGAAGCCTGTTG
flbC	BBA_03181	C_2H_2 conidiation transcription factor FlbC	TCCATCTCCAACTTGCTGGGTCTC / GGCGGCGTAGGCGGAAGG
flbD	BBA_07259	MYB conidiophore development protein FlbD	CGGCAAGCGATGGGCAGAGATTG/ CGAGCAAGGTGACGGTAGAGGTG
flbE	BBA_01716	Conidiophore development protein FlbE	CAGACGATGAGACAGAGA / GGGCTTTATATGCGAGTAG
brlA	BBA_07544	C_2H_2 conidiation transcription factor BrlA	GACCAGTTCAACAGACAAG / CAGTAATCTTCGTGCTTCTC
abaA	BBA_00300	Conidiation factor AbaA	GCAAGTCTCCAGCCATAT / CTCCTCTTCGTCATAGTAGTC
wetA	BBA_06126	Conidial maturation factor WetA	CGCAGACGAATTTGACTT / GCTGGTGGTTGAATACAT
vosA	BBA_01023	Velvet protein VosA	GGACAGACGAGTGATTGA / GGCATATACGACGCATCT
**Involved in hyphal septation**			
apsA	BBA_05547	Cell division/GTP binding protein ApsA	CTCTGCTCTGCTTCATAG / TTCTCGGTACGGTAGTTC
apsB	BBA_06894	Cell division/GTP binding protein ApsB	TTACCGACAACATTGAAC / GGATGAAGAAGACACAAG
apsC	BBA_04748	Cell division control protein 12 ApsC	GGAGAAGTTCTTCAAGGT / AGGAGTCACGATTGTTAA
chsA	BBA_03236	Class 2 chitin synthetase ChsA	GTGTCTTACTGATTGCTA / ATAATGATGGCGTAGATG
chsB	BBA_04667	Chitin synthetase 3a ChsB	CTCTGCCAATGTCACCAAG / TCAATGTCTTCCTGCTCCTT
chsC	BBA_03590	Chitin synthetase 1 ChsC	CGATCTGAACCAGCACAA / ATGACGGAGACGATGAGT
bud3	BBA_03175	RhoGEF domain-containing protein Bud3	GATAATCCTATAATACGACTC / GTAGACATCATTGACAAG
bud4	BBA_01075	GTP binding protein Bud4	GCGTCATCATCACCAGAA / TAATCTCATCATCCTCATCATACA
rho4	BBA_03155	Ras family protein Rho4	CACATACTCCAAGAAGATT / AAGACGGTAGGAACATAT
sepH	BBA_03315	Cell division control protein SepH	AACTGCCTCAACATCATC / TCATATACACGCCAACAAG

* Gene accession codes in *B. bassiana* genome under the NCBI accession NL_ADAH00000000 (Xiao *et al*., 2012).
